# Absence of hepatitis E virus RNA in bovines from Portugal

**DOI:** 10.1007/s11259-025-10839-0

**Published:** 2025-07-30

**Authors:** Sérgio Santos-Silva, Josman D. Palmeira, Helena Ferreira, Jesús L. Romalde, Helena M. R. Gonçalves, Wim H. M. Van der Poel, Maria S. J. Nascimento, António Rivero-Juarez, João R. Mesquita

**Affiliations:** 1 School of Medicine and Biomedical Sciences (ICBAS), University of Porto, Porto, Portugal; 2 UCIBIO—Applied Molecular Biosciences Unit, University of Porto, Porto, Portugal; 3 Associate Laboratory i4HB, Institute for Health and Bioeconomy, University of Porto, Porto, Portugal; 4 Microbiology Laboratory, Biological Sciences Department, Faculty, Pharmacy of University of Porto, Porto, Portugal; 5 Cross-disciplinary Research Center in Environmental Technologies (CRETUS), Department of Microbiology and Parasitology, CIBUS-Faculty of Biology, Universidad de Santiago de Compostela, Santiago de Compostela, Spain; 6 Department of Chemistry and Biochemistry, Faculty of Sciences, LAQV, REQUIMTE, University of Porto, Porto, Portugal; 7 Infectious Diseases Epidemiology, Wageningen University, Wageningen, The Netherlands; 8 Department Virology & Molecular Biology, Wageningen Bioveterinary Research, Lelystad, The Netherlands; 9 Faculty of Pharmacy, University of Porto (FFUP), Porto, Portugal; 10 Unit of Infectious Diseases, Clinical Virology and Zoonoses, Instituto Maimonides de Investigación Biomédica de Córdoba (IMIBIC), Hospital Universitario Reina Sofia, Universidad de Córdoba (UCO), Cordoba, Spain; 11 Center for Biomedical Research Network (CIBER) in Infectious Diseases, Health Institute Carlos III, Madrid, Spain; 12 Centre for Animal Science Studies (CECA), Institute for Science, Technology and Environment (ICETA), University of Porto, Porto, Portugal; 13 Associate Laboratory for Animal and Veterinary Science (AL4AnimalS), Lisbon, Portugal

**Keywords:** HEV, One health, Bovine, Zoonosis, Epidemiology

## Abstract

Hepatitis E virus (HEV) is widely recognized as an emerging public health issue in developed countries, with most infections linked to foodborne transmission of genotype HEV-3. This zoonotic genotype can infect a diverse range of mammalian species, including bovine, with pigs serving as the primary reservoir. The aim of the present study was to investigate the occurrence, circulation, and the potential of HEV infection among bovines in Portugal. Stool samples were collected from 166 bovines raised on extensive and intensive farms in Portugal, from June one to July 31, 2015. For the detection of HEV RNA a nested broad-spectrum RT-PCR targeting the ORF1 region was used. HEV RNA was not detected in any of the fecal samples analyzed. Although no HEV RNA was detected in bovine fecal samples, spiking of the samples with mengovirus demonstrated an acceptable RNA recovery rate, ensuring the reliability of RNA extraction and subsequent molecular analysis performed. Further research could provide additional insights into the factors influencing HEV transmission dynamics in bovines and its potential implications for public health.

## Introduction

Hepatitis E virus (HEV) is a single-stranded RNA virus with a genome length of 6.4–7.3 kb with three partially overlapping open reading frames (ORF1, ORF2, and ORF3). Viral particles are 27–34 nm in diameter, nonenveloped in feces and bile, and membrane-associated quasi-enveloped in blood (Takahashi et al. 2010; Kamar et al. 2012; Debing et al. 2016; Nagashima et al. 2017). HEV belongs to the family *Hepeviridae*, subfamily *Orthohepevirinae* (Purdy et al. 2022). *Paslahepevirus* genus includes the species *P. balayani*, which comprises eight genotypes (HEV-1 to HEV-8). HEV-1 and HEV-2 infect only humans, HEV-3, HEV-4, and HEV-7 infect both humans and animals, while HEV-5, HEV-6, and HEV-8 are restricted to animals (Smith et al. 2020). HEV is the only human hepatitis virus with confirmed zoonotic transmission and ranks sixth among 887 wildlife viruses for spillover potential (Grange et al. 2021).

Pigs are the primary reservoir for HEV-3 and HEV-4, which are responsible for most zoonotic cases in industrialized countries. Wild boars have also been recognized as important wildlife reservoirs, contributing to environmental contamination and spillover risk through hunting and meat consumption (Pavio et al. 2015; Salines et al. 2017). Human infection is mainly associated with the consumption of undercooked pork or wild boar meat, but contact with infected animals or contaminated environments can also facilitate transmission.

In recent years, increasing attention has been directed toward the potential role of bovines in the HEV transmission cycle. Although some studies have demonstrated evidence of HEV in bovine (Yu et al. 2009; Huang et al. 2016; Yan et al. 2016; Go et al. 2019; Mesquita et al. 2019; Rahmani et al. 2020; Sayed et al. 2020; Bastos et al. 2022), the significance of cattle as reservoirs remains unclear. The presence of HEV RNA has been detected in bovine milk and liver samples, suggesting that the consumption of raw milk or meat from infected bovine may pose a potential risk of infection to humans (Huang et al. 2016; Go et al. 2019; Bastos et al. 2022; Turlewicz-Podbielska et al. 2023; Zahmanova et al. 2024). However, the prevalence of HEV in bovine varies significantly between regions, with some studies finding no evidence of infection in certain areas (Geng et al. 2019). Furthermore, the seroprevalence of anti-HEV antibodies in bovine has been reported at varying levels, indicating exposure to HEV, but without consistent confirmation of active infection (Yugo et al. 2019; Pugliese et al. 2021). The detection of HEV in milk raises concerns for foodborne transmission, particularly through the consumption of raw dairy products (Huang et al. 2016). These findings suggest that bovines may act as incidental hosts or secondary reservoirs, warranting further investigation. Furthermore, while *P. balayani* is the main zoonotic hepatitis E virus, the identification of *Rocahepevirus ratti* in human cases has expanded concern within the *Hepeviridae* family (Sridhar et al. 2018, 2022; Andonov et al. 2019). Although rodents are the primary hosts, many human infections lack confirmed rodent contact, suggesting possible involvement of other animals or environmental sources (Reuter et al. 2020). Given emerging evidence of HEV in bovines and the potential for non-traditional reservoirs, it is important to include this virus in molecular surveillance alongside *P. balayani*.

Taking everything into account and focusing on fecal material to detect HEV RNA, the aim of this study was to investigate the presence of HEV and other zoonotic hepeviruses, such as *Rocahepevirus*, in stool samples from bovines in Portugal.

## Materials and methods

### Sample collection

**Fig. 1:**
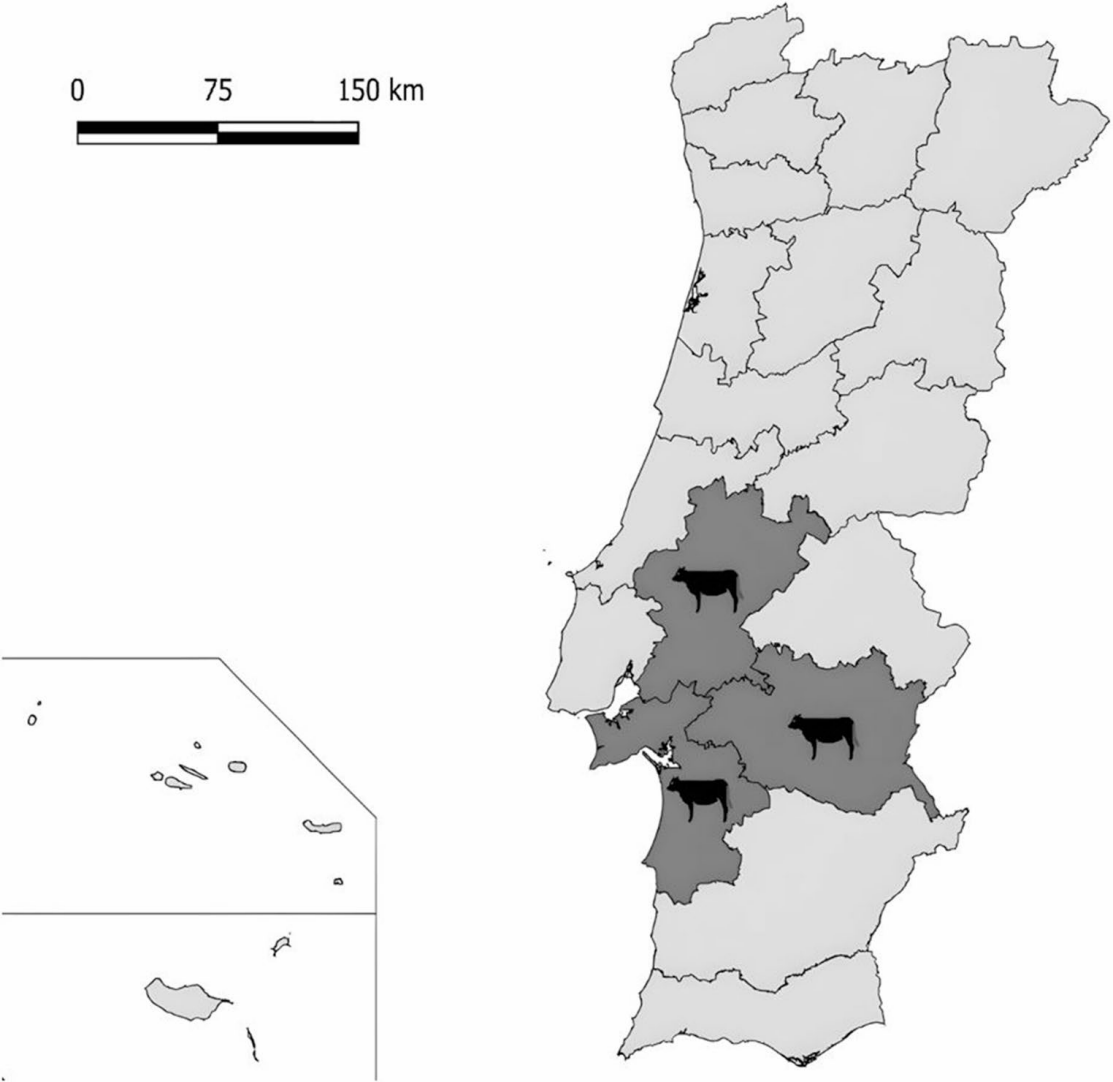
Geographical distribution of sampled bovines in Portugal

This retrospective study included bovine stool samples originally collected in June/July 2015 from seven farms located in the central/southern region of Portugal, namely Santarém, Setúbal and Évora districts, as part of a previous study with a different research goal (Gomes-Gonçalves et al. 2023). Of the total bovines sampled (*n* = 166), 142 were from intensive and 24 from extensive farming systems, originating from seven different farms. All stool samples were preserved at − 80 °C until they were reused in the present study (Fig. 1).

### Nucleic acid extraction

Stool suspensions (10%) were prepared in phosphate-buffered saline (pH 7.2) and centrifuged for 5 min at 8000× *g*. One of the main obstacles in viral detection is the low efficiency of viral RNA extraction. This limitation can lead to false-negative results due to poor RNA recovery, particularly when the sample has a low viral load. To address this issue, process control is commonly used to monitor RNA extraction efficiency (Ahmed et al. 2022). These inefficiencies have been identified as a significant factor contributing to the persistent challenge of detecting at least half of foodborne viral outbreaks (Stals et al. 2012). To address this issue, before RNA extraction, 10 µL of mengovirus (MeV) clone vMC0 (final concentration of 10⁵ genomic copies (GC)/mL) was added to the first stool sample in each batch of 12 samples processed using the QIAcube^®^ automated platform as a control for virus extraction (Costafreda et al. 2006). RNA was extracted and purified from 200 µL of the clarified supernatants using the QIAamp Viral Mini Kit (Qiagen, Hilden, Germany) on the QIAcube^®^ automated platform (Qiagen), following the instructions from the manufacturer. Eluted DNA and RNA were stored at − 80 °C in RNase-free water.

### Detection of mengovirus and determination of extraction efficiency

Detection of mengovirus (MeV clone vMC0) was performed using RT-qPCR, as outlined in a previous study (Costafreda et al. 2006). Reactions were run on a CFX Connect Real-Time PCR Detection System (Bio-Rad, Hercules, CA, USA) thermocycler using with the Xpert OneStep Fast Probe (GRiSP^®^, Porto, Portugal), following the instructions provided by the manufacturer. The thermal cycling conditions for the RT-qPCR reaction included an initial reverse transcription (RT) step at 50 °C for 15 min, followed by a simultaneous step for reverse transcriptase inactivation and the initial denaturation of cDNA at 95 °C for 5 min. Consequently, 40 cycles of amplification were carried out, involving denaturation at 95 °C for 5 s and annealing/extension at 60 °C for 20 s. Afterwards, results were analyzed using the CFX Maestro 1.0 Software version 4.0.2325.0418 (Bio-Rad, Hercules, CA, USA). Extraction efficiencies were calculated in accordance with ISO 15216-1:2017 (International Organization for Standardization 2017). The effectiveness or recovery rate of MeV was classified as unacceptable (< 1%), acceptable (1–10%), or good (> 10%)(International Organization for Standardization 2017).

To estimate the likelihood of false negatives due to the extraction recovery efficiency observed in this study, a power calculation assuming viral loads ranging from 10 to 100 copies was performed. The number of recovered viral copies were modeled as a Poisson distribution with a mean (λ) calculated by multiplying the viral load by the recovery efficiency (3.1%). The probability of detecting at least one viral copy (true positive) was then calculated as 1 − e^−λ^, where 𝑒 is the base of the natural logarithm.

### Detection of HEV

All RNA extracts were first screened individually using a broad-spectrum real-time RT-PCR (RT-qPCR) assay targeting the ORF3 region, using primers and a TaqMan probe as described previously (Jothikumar et al. 2006). The RT-qPCR was conducted with the iTaq Universal Probes One-Step Kit (Bio-Rad Laboratories, USA) in a total reaction volume of 20 µL, run on a CFX Connect Real-Time thermocycler (Bio-Rad Laboratories, USA). The thermal cycling protocol started with reverse transcription at 50 °C for 10 min, followed by a combined reverse transcriptase inactivation and initial cDNA denaturation at 95 °C for 3 min. This was followed by 45 cycles of amplification, each consisting of denaturation at 95 °C for 15 s and annealing/extension at 55 °C for 15 s.

Further HEV RNA detection was performed for all samples individually using a broad-spectrum nested RT-PCR assay targeting a 331–334 bp fragment in the ORF1 region, which is capable of detecting both *Paslahepevirus balayani* and *Rocahepevirus ratti* (Johne et al. 2010). In the first PCR round, primers HEV-cs and HEV-cas were used, while HEV-csn and HEV-casn were employed for the second round (Johne et al. 2010). All PCR reactions were conducted on a T100 thermocycler (Bio-Rad). For the first PCR round, the Xpert One-Step RT-PCR kit (GriSP^®^, Porto, Portugal) was used, followed by the Xpert Fast Hotstart Mastermix 2x with dye (GriSP^®^, Porto, Portugal) in the second round.

Thermocycling for the first round involved cDNA synthesis at 45 °C for 15 min, an initial denaturation at 95 °C for 3 min, followed by 40 cycles of 95 °C for 10 s, 50 °C for 10 s (annealing), and 72 °C for 15 s (extension), with a final extension at 72 °C for 10 min. In the second round, an initial denaturation at 95 °C for 3 min was followed by 40 cycles of 95 °C for 15 s, 50 °C for 15 s (annealing), and 72 °C for 2 s (extension), with a final extension at 72 °C for 10 min.

The PCR products were visualized by electrophoresis on a 1% agarose gel stained with Xpert Green Safe DNA gel dye (GriSP^®^, Porto, Portugal) and run at 120 V for 30 min. The results were confirmed using a UV transilluminator.

## Results and discussion

In the present study, HEV was not detected in any of the analyzed stool samples, indicating either its absence in the tested bovine population or that the virus was not present in detectable quantities in the fecal matter under the conditions of this study.

To assess the quality and reliability of the nucleic acid extraction process, we evaluated the recovery of the spiked process control (MeV). The recovery rates of MeV varied between 1.05% and 5.04%, with an overall average of 3.08%, falling within an acceptable range. These results suggest that the nucleic acid extraction procedure was efficient and that the samples were processed adequately, ensuring the reliability of the findings. The variation in recovery rates may reflect inherent differences in the fecal sample matrix, but overall, the extraction process yielded results within an acceptable range, confirming that the absence of HEV RNA in the stools was not due to methodological issues. Nonetheless, it is important to acknowledge that the stool samples had been stored for some time at −80 °C. Moreover, power calculation indicates that the probability of detection increases from approximately 27% at 10 copies to 96% at 100 copies, indicating reliable detection in samples with moderate to high viral loads despite low recovery efficiency. Furthermore, although the samples were collected in 2015, current HEV shedding levels in Portuguese cattle may differ. Nevertheless, these data remain informative, offering a valuable baseline for assessing temporal trends and guiding future surveillance efforts in regions with limited data. Despite all this, potential RT-PCR inhibition was not directly assessed in this study, and the relatively low MeV recovery rates may also partially reflect inhibitory effects inherent to the fecal matrix. Future studies could benefit from incorporating internal inhibition controls across different sample matrices to further validate assay performance.

The presence of HEV RNA in bovines has been reported in several regions of the world (Yu et al. 2009; Yan et al. 2016; Go et al. 2019; Mesquita et al. 2019; Bastos et al. 2022), including Europe (Rahmani et al. 2020). Despite a report on HEV in a study from Romania (Rahmani et al. 2020), the study did not determine the HEV genotype.

Moreover, to date, several studies have investigated the presence of HEV RNA in bovines using fecal samples, serum, milk, and other biological matrices, yielding variable results. While some studies reported no detection of HEV RNA in bovine fecal samples (Reuter et al. 2009; Forgách et al. 2010; Prpić et al. 2015; Tritz et al. 2018), positive detections have been reported in other sample types such as milk and serum, with rates ranging from 0.19 to 37.14%, depending on the sample type (milk, serum), geographic location, and the population studied (bovines, cattle, or cows) (Yu et al. 2009; Huang et al. 2016; Yan et al. 2016; Mesquita et al. 2019; Bastos et al. 2022). This highlights the variability in HEV RNA detection based on the biological matrix analyzed, which may influence the sensitivity of surveillance efforts. Furthermore, molecular characterization from these studies has identified diverse HEV genotypes and subtypes in bovine, such as HEV-4 and its subtypes 4 d and 4 h in China (Huang et al. 2016; Yan et al. 2016), and HEV-3 in South America (Bastos et al. 2022). In the present study, HEV RNA was not detected in any of the stool samples collected from 166 bovines across seven farms in the central and southern regions of Portugal. This finding is consistent with studies that have reported the absence of HEV RNA in bovines from other European countries (Reuter et al. 2009; Forgách et al. 2010; Vercouter et al. 2018). However, reports from other regions, such as China, have frequently identified HEV RNA in bovines, particularly in serum samples (Yu et al. 2009; Huang et al. 2016; Yan et al. 2016). These discrepancies may be attributed to variations in sampling strategies, geographic differences in HEV prevalence, and the biological matrices analyzed. Altogether, these findings underscore the need for further research to assess HEV prevalence in bovine populations across diverse settings and sample types.

The present study represents an important first step in understanding HEV circulation in bovine in Portugal. However, it is worth noting that while molecular evidence of HEV infection was not found in the fecal samples analyzed, type of samples chosen because of their non-invasive collection and relevance for environmental shedding, continued surveillance and expanded studies, including other sample types such as liver and serum, and direct sampling at farm levels, using stool samples that are as fresh as possible, are warranted to gain a comprehensive understanding of HEV prevalence and transmission dynamics in bovines in the region. Future research should also consider conducting more comprehensive epidemiological surveys, including larger sample sizes, diverse geographic locations, and different farming systems, to better assess potential risk factors for HEV infection and, if necessary, implement appropriate preventive measures to mitigate its spread within livestock populations.

## Data Availability

No datasets were generated or analysed during the current study.
